# On Feigned and Factitious Diseases, Chiefly of Soldiers and Seamen

**Published:** 1844-01-01

**Authors:** 


### On Feigned and Factitious Diseases, chiefly of Soldiers and Seamen. By *Hector Gavin*, M.D. &c.

As all animals have been classed into devouring and devoured, man holding a somewhat commanding position in the first of these; so may human beings be considered under the two comprehensive heads of deceivers and deceived. Which is the most numerous of the two last, it would not perhaps be difficult to decide, but the question is no further pertinent to the subject of our remarks than as succinctly evincing the antagonism that has, and ever will continue to exist, between the general weal and individual selfishness. Through the whole economy of the political and social machinery, this antagonism will be found in as constant relation of action as the extensor and flexor muscles of the body. So long as it exists in moral harmony it may be the source of all virtue, and can be conducive to good only; while an undue force of one of the principles alluded to, will as obviously lead to deception and crime. In proportion then as either force acts, will depend our utility in life, and the amount of reputation that posterity may allow us.

Though class legislation has undoubtedly greatly improved, a time was when the health and comfort of our seamen and soldiers would seem to have been little considered or cared for by authority. Great, marked, and beneficial, is the change that has taken place in this respect. The evils of gross neglect, and bad regulation, rendered the old system intolerable to many. This has not been dwelt upon by the author of the work the title of which heads our observations. He refers indeed to the effects of the Conscription in France, but what was as bad as the conscription, our own old pressing and recruiting system, no less than the modes once in vogue for enforcing discipline, were equally prolific sources of unhappiness, and of expedients to fly from it. Malingering, skulking (or sham-Abrahaming) Dr. Gavin has shewn to be of very ancient origin. It requires no great stretch of retrospection to recal days when the corrective principle of Draco was dominant, not only in our general criminal code, but in our fleets and armies.

Castigatque, auditque dolos, subigitque fateri, was as much the rule with naval and military captains as with that excellent, but somewhat summary Judge, Rhadamanthus. Punishment was too often as capricious in its cause, as wanton and cruel in its degree and extent. No wonder that this should have been the case with the men, when the officers were liable to the outrage of brutal vituperation and retort, as well as to become the victims of malignant tyranny. The pages of Smollett and others abound in examples, as do those of able writers in our own day. The surgeons and surgeons' mates, in what in many respects is the "ow'r true tale" of Roderick Random, afford a lively idea, but not a very agreeable one, of the treatment that gentlemen of the navy were liable to in the reign of George II. One is represented as chained to the poop, by order of his tyrannical commander, during a terrific sea engagement, while another resorts to a suicidal expedient, in order to escape from his malicious persecutors. If we are not better at the core than our ancestors, we may certainly claim a higher standard of decorum, and all that the first of our lexicographers understood by civility in its amplest sense. During the last thirty years even, to say nothing of a hundred, a glance at our current literature may satisfy even a desultory observer of the correctness of this assertion. To those who have never quitted their native shores, this may not be so obvious, but it is very observable on the surface of manners, to a person returning to England after a long absence. He recognises an improved tone, as regards manners, appearance and language, in the lower orders. Much of this may be owing to legislation, but the great Archimedes' screw of improvement that impels, is the progress of knowledge, which legislation never precedes but follows *haud passibus æquis*. Of this truth, or rather truism, numerous examples might be given. One will suffice. What is called the Peel bill for the reformation of the criminal code had, long before he appeared on the stage of affairs, been demanded by the spirit of the age, and Sir Samuel Romilly had devoted a great portion of his valuable life to demonstrate its necessity, when the more fortunate politician of the two was comparatively little known. Laws do much, but when not backed by public opinion, they are frequently weak or inoperative for good. Concurrent they are irresistible for good or evil. It was public opinion, by little and little gathering force from its feeders, a rivulet here and a streamlet there, until it formed one strong and broad current, carried reform upon its waves, into the army and navy. Many of the causes of malingering then, such as vexatious and harsh controul, and cruel discipline, have ceased. We have no longer the hectoring bearing and abusive language of days gone by, when the vituperative epithet and the sharp rattan followed each other as surely and quickly as the thunder does the flash of lightning.

The hope of advantage from pension, &c. no doubt is a fruitful source of malingering, but when malingering continues to prevail to such an extent as it has been proved to do, is there not ground for apprehension that some defects still cling to our naval and military system in its internal working? We acknowledge, and gratefully, the immense amelioration it has undergone, even within our own recollection. Not only is the moral improvement of the men sedulously promoted, but their comfort in various ways, and even their amusements, are carefully looked to. Time was when to relieve the tedium of a soldier's life, cleaning accoutrements, and furbishing firelocks and bayonets were deemed the sovereign resource. To be sure, notwithstanding these delectable pursuits, which, repeated day after day, *are* apt to make the soldier hate the very look of his arms, that ought rather to be his pride as the signs of his power—the hospital became a preferable lounge to the barrack or parade, whenever the regimental surgeon could be imposed upon. Man was in those days considered so completely a machine, that mere physical friction came to be looked upon as a sort of necessary process, just as if the polish and brightness of his firelock and bayonet passed into his humanity. So that his arms and spine were undergoing fatigue, he was out of harm's way. The authorities were of the famous orator's opinion, that action, action, action, was everything. Was there a rumour of discontent or unhappiness among the men? Set them to pipe-claying, to learn the goose-step—anything, in short, so that their hands and feet have as much friction as their arms. Provided there was enough and to spare of this gearing and polishing in barracks the very perfection of a soldier's morale was considered as attained. A colonel of the old stamp would have stared at the ridiculous notion of a soldier deriving entertainment from a book, or amusement from a game at rackets or skittles. Acknowledging then how much has been done for bettering the condition of our soldiers and sailors, let us not lay the flattering unction to our souls that all is perfect. Much depends upon the conduct of the officers. To this, and the most careful attention in recruiting and drafting, do we attribute it, that some regiments are models of good government. That others are sadly the reverse is almost wholly the fault of perverse or blundering commanding officers. That men, remarkable either for bad temper or incapacity, should be allowed, on irrefragable proofs of either, to retain command, is one of the effects of our aristocratic tendencies in all departments of government. The experiment has had its full swing, and may some day produce results little dreamed of. We know what its effect was in France! A great deal depends upon the personal bearing of officers towards the men. Without the slightest compromise the behests of superiors may be sweetened by common urbanity. When avoidable, the men ought, especially in hot climates, to be put as little as possible, on what are called fatigue duties. Sometimes in a Tropical latitude a trifle may rouse the soldier to acts of frenzy. We recollect an instance of one who shot his officer dead at the main guard, because he was refused (not by the poor officer but his native servant) a glass of water. In another regiment, two murders of the most deliberate kind occurred in Fort William within a few days of each other, and the only apparent reason for their perpetration was the annoyance of the unhappy men at having been kept longer than they deemed just or necessary at fatigue duty during warm weather—there were six lives lost in consequence of what may be deemed a passing trumpery irritation. Nothing is trifling, however, that has the slightest tendency to excite the soldier to such fearful deeds, and that he is liable to be so excited at times, in a hot climate, is a melancholy truth of which there are many awful examples on record. In no instance of such sudden outbreaks within our recollection was the plea of derangement ever made out to the satisfaction of the court, when resorted to. The precedent of monomania in mitigation of consequences would by many be deemed a hazardous one in the army and navy. It is a double-edged question whichever way we view it. To deny the existence of moral insanity, because there may be no obvious intellectual flaw, would be to fly in the face of evidence. To be guarded against its simulation, or being made a convenient plea, is another matter.

The origin of Dr. Gavin's work is another and successful instance that to rouse talent to honourable exertion, a generous stimulus is sometimes required. The work itself appeared some years ago in a less extended form, in competition with many others for a prize proposed by Sir George Ballingall, for the best Essay " On the best Classification of the Feigned and Factitious Diseases of Soldiers and Seamen, &c. &c." It is very properly dedicated to one whom the profession, individually or collectively, cannot too much honour, we mean Sir James M'Grigor. Nearly in our author's own words, the direct scope of the work (which ought to be in every practitioner's hands, more especially the Medical Staff of the Navy and Army,) may be said to be, to prevent the honourable physician being made the dupe of the artful impostor, or guard him against judging too harshly in doubtful cases, and unjustly punishing the innocent, more especially from being himself the instrument of punishment in presumed cases of malingering. The importance of the subject will at once be admitted by the political economist, when it is borne in mind what an immense saving to the country may be made by the sharp-sighted and experienced surgeon who prevents the drones of the service from plundering, and is constantly on the watch to baulk " the Artful Dodgers," who on the pretence of injury or disease, when neither really exists, wish to cheat the State by quitting its service prematurely, in getting smuggled, if they can, into the pension list, which ought to be sacred to its proper objects, instead of an Alsatia for knaves and malingerers to creep into. As the author has shewn, disease has been simulated in every age, and by all classes of society. It is not confined to the ranks, for generals and colonels can malinger occasionally just as well as their inferiors. There have been even commissioned officers who have had the good taste to boast that they had *done* the Doctor! Were it only in self-defence and to prevent such dishonourable exultation, the medical officer ought always to be on his guard when he is applied to for a sick certificate. No matter what may be the rank or position of the applicant, before granting such a certificate, the surgeon should be convinced beyond all doubt that there are good grounds for it. He ought to be above the suspicion of being open to wheedling on such a point, even by his own brother, were he to attempt it.

It is not once that we have heard such expressions as "O, very well, since you will not oblige me, I will go to Mr. ———, he will give me a certificate without all this fuss." Supposing a medical officer grants a certificate, although not convinced in his own mind of its being absolutely a case requiring one; what is this but knowingly to give the stamp of truth to a lie?

Sallust, in his admirable character of Catiline, observes that, among his other accomplishments, he was "audax, subdolus, varius, cujuslibet rei simulator ac dissimulator.* It is as a simulator that the malingerer shines with Catiline-like flexibility and ability, as our author well illustrates, for dissimulated or concealed diseases do not fall within the province of his work. The motives that induce soldiers and sailors to simulate diseases, though many and various, are thus briefly but comprehensively classed.

"To obtain their discharge from the service, with or without a pension, &c.; to avoid the performance of the duties which are imposed upon them;—to escape some particular service which is disagreeable to them, or to obtain some other that is agreeable;—to obtain, *or prevent*, their removal from one climate or station to another;—to obtain the ease and comforts of an hospital, &c.—sometimes, though rarely, to bring blame or punishment on an individual whom they dislike;—to avoid an apprehended or adjudged punishment;—to excite compassion or interest;—the hope of gain, &c." 12.

Revenge induces some to magnify slight ailments. This has often occurred in India, where it has even induced the simulation of death. In one instance, a young writer gave a slap to a native servant, who fell down apparently dead. In a great fright he had the body carried over instantly in his palankin to the Honourable Company's Dispensary, where the application of a magnum bottle of very potent liquor ammoniæ to the dead man's nostrils, so astonished his olfactories, that he instantly jumped up and bolted! Indeed all classes in India are adepts at simulation, and servants, when they want a holiday, crave leave on the plea of the sudden death of father, mother, or child, which father, mother, or child, or each and all of them will be found, if a record has been kept, to have, like a once noted amateur actor, who was sometimes required to do so on the stage—died over and over again.

"As diseases are feigned for a variety of purposes, so the character of the assumed disability is calculated to suit the occasion. If a soldier wishes to escape or delay punishment, to evade duty of any kind, more especially that of embarking for foreign service, an acute disease is simulated. If, however, the design be to obtain a discharge, with or without a pension, an infirmity of another class is feigned—one which possesses a chronic, incurable character, calculated, if possible, to excite pity and commiseration. With reference to the first class, Hennen remarks, that there are some diseases, the symptoms of which are so obvious to a well-informed medical man, who watches them closely, and at times when he is not suspected, that no artifice of those who pretend to labour under them can deceive him." 19.

---

* According to Commentators *Simulator* is dicitur qui id esse ostendit, quod non est; *Dissimulator* contra, qui id non esse quod est.

It is of importance to remember that, " In diseases characterised by obscure, variable, or uncertain symptoms, much care should be taken not to be misled; for every practitioner knows that there are some diseases in which there is no change of pulse, or alteration of the natural colour or temperature of skin, or any evident derangement of functions of the organ implicated, to indicate their existence. There are also other diseases, whose symptoms may be imitated by the effects produced by certain drugs, or by the use of certain external applications. It is, therefore, well remarked, that 'an intimate knowledge of the anatomy, physiology, and pathology of the human body, and of the effects of the articles of the Materia Medica, is consequently essential to the medical practitioner, to enable him, in such cases, to obviate false conclusions and detect imposture.' "

There can be no doubt but the diminished number of deceptions, both in the army and navy, is attributable, firstly, to the ameliorated condition of the men; and, secondly, to improved medical vigilance. The Irish are stated to be the most numerous and expert at counterfeiting disease, next comes the Lowland Scotchman, who makes up in obstinacy what he wants in address; and of the English soldier, it is said to be his least vice. The simulator finds it difficult to give a consistent account of his alleged disability. " By a little address the surgeon can lead him to enumerate incompatible symptoms, or greatly to exaggerate unimportant lesions. *He is constantly prone to overact his part.*" This should never be forgotten, as much of our detective potentiality depends upon it. An impostor too, we learn, unprepared with a set of symptoms, has been detected by the abrupt question, " what is the matter with you ? " The nonplussing effect must be rather comical after " the fellow having been led by the leading questions of the medical officer to enumerate symptoms of disease from head to foot." The surgeon should beware of having his patience tired out by the malingerer, or of letting him go with the remark, " He is an useless fellow—a better man may be obtained in his place." Our author very justly observes—" Nothing can be more fallacious than this doctrine; for no sooner does one of the impostors succeed, than two or three more are sure to follow his example, in the hope of obtaining their discharge by pursuing the same plan." In examining inefficient men, the medical officer is warned to pay due regard to their claims, while he devotes the requisite degree of attention to the public interest. Great care should be taken to distinguish between temporary and permanent disabilities for service. Disabled men should be examined without their clothes. Violent measures and language ought always to be avoided. Intimidation can effect nothing with a determined malingerer. " In no instance should means be employed to detect a suspected person which a medical officer would regret having used, were the alleged disability to prove real." This is an excellent injunction. In cases of feigned disabilities of a chronic character, as palsy, contractions, &c., we are advised rarely or never to employ medical means. The rules laid down by our author for determining the reality or otherwise of a disease are exceedingly good; but to state them at length would press too much on our limits. Stress is very properly laid on the care necessary to discriminate beween reality and simulation. An instance of the vital necessity of great caution in this, fell under our own observation. In a regiment of native infantry, doing duty in the field on a frontier part in Bengal, a Classee or tent pitcher was the bearer of a note from the adjutant to the medical officer in charge, (a young man,) stating that he wrote at the request of the commanding officer, who, as well as himself, was convinced that the Classee was shamming in order to evade duty; that they sent the man to be examined, and if he (the medical officer) agreed in opinion with them, as they doubted not he would, care should be taken to have him flogged summarily. The Classee took the note to the medical officer just as he was going the evening rounds of his side. The man was a strong hale stout looking fellow, of about 35, but with a somewhat heavy expression of countenance. He complained merely of slight headache, and feeling languid. The man looked the picture of health—his tongue was clean—his skin cool, but his pulse was a little slower than the average. The surgeon felt that there was something in the case that required further inquiry, and especially that time should be taken to test whether the slowness of pulse was constitutional or symptomatic. He was ordered to take some opening medicine, and a note was written to the adjutant to say, that, to the best of the surgeon's judgment, founded upon a brief inspection, the man was not well. The poor fellow dropped down suddenly dead in the night, no doubt from apoplexy. No autopsy was allowed by the friends. Perhaps a good large bleeding might have saved the poor fellow. It was, at any rate, consolatory to all parties next day, that he had been sent into hospital instead of being returned bearing a note to the adjutant, that there appeared to be nothing the matter with him, in which case he would have assuredly been punished,* and his death would have been afterwards attributed to such punishment, to the dismay of all parties concerned. Commanding officers, or their staff, ought never to attempt thus to prejudge a case, or to try and biass the medical officer, as it is his province alone to decide calmly and deliberately whether a man be shamming disease or not. Many a man may be very ill, who somehow cannot state his case clearly, though he may appear stout and well. It was so with the Classee. He seemed indifferent in regard to whether he was thought ill or not.

We cannot conform to the rule of Percy and Laurent 'that we should always incline to consider the case as one of simulation rather than of reality.' Supposing the rule acted upon in the Classee's case, might it not have led to his being flogged? In commenting upon this rule, Dr. Gavin very justly and with good feeling states, "that we should simply endeavour to discover the truth, without being afraid to find a man guilty, and without entertaining a wish that the person under examination should be detected as an impostor."

Such a classification as Dr. Ballingall sought for, or one that would enable us to proportion the rate of pension to be given to soldiers who are to be discharged on account of disease, cannot be obtained, our author is of opinion, by following the arrangement of any nosologist. Accordingly he submits the following:—

---

* No summary punishment is now permitted.

### CLASSIFICATION OF FEIGNED AND FACTITIOUS DISEASES,

*Founded on our Means of Diagnosis, namely, on those Symptoms which are referrible to the Feelings of the Patient, and those which are cognisable by the Senses, or acquired Information of the Physician.*

Pain.
Nyctalopia.
Hemeralopia.
Amaurosis.
Myopia.
Presbyopia.
Amblyopia.
Strabismus.
Nictitation, Blepharospasmus.
Deafness.
Aphonia.
Dumbness.
Deaf-Dumbness.
Stammering.
Insensibility, Coma.
  Lethargy and Somnolency.
Somnambulism.
Vertigo, Cephalalgia.
Hysteria.
Insanity.
  Dementia, Imbecility.
  Mania.
  Monomania.
    Erreur de Sentiment.
  Moral insanity.
Nostalgia.
Epilepsy.
Convulsions.
Chorea.
Catalepsy, Cataleptic Extasy.
Paralysis.
  Hemiplegia.
  Paraplegia.
  Local Paralysis.
    Ptosis.
Shaking Palsy.
Chronic Rheumatism.
Hæmaturia, Coloration of the Urine.
Phthisis.
Abdominal Tumour.
  Physconia.
  Tympanitis.
Peritonitis, Gastritis.
Syncope.
Palpitation.
Hypertrophy of the Heart, and Pericarditis.
Excited circulation.
Diminished circulation.
Asthma.
Acute Rheumatism.
Lumbago.
Sciatica.
Dysuria.
Incontinence of Urine.
——— the Fæces.
Ischuria.
Lameness—Voluntary Limping.
Disease of the Loins, from Hurts, Sprains, &c.
Chronic Hepatitis, Hepatalgia.
  Acute ———
Intermittent Fever.
Continued ———
Dyspnœa.
Pneumonia.
Vomiting.
Pyrosis.
Gastralgia, Gastrodynia.
Dysphagia.
Dyspepsia.
Colic.
Malformations, Deformities.
  Lateral curvature of the Spine.
  Gibbosity.
  Obstipation.
  Contractions of the limbs.
  ——— of the shoulder-joint.
  ——— of the elbow-joint.
  ——— of the fingers.
  ——— of the thigh.
  ——— of the knee-joint.
  ——— of the ankle-joint.
Malposition of the toes.
Epistaxis.
Hæmatemesis.
Hæmoptysis.
  Pompholyx.
  Porrigo.
  Erysipelas.
  Scabies.
  Variola.
Gout.
Stricture of the Urethra.
Otorrhœa.
Cancer.
Fistula in Ano, in Perinæo.
Ozœna.
Fætid Perspiration.
——— Breath.

Ophthalmia.
  Tarsi.
  Membranarum Conjunctivitis.
White-swelling.
Scrofula.
Lupus.
Ulcers.
Factitious Wounds.
  Loss of teeth.
Fictitious Wounds.
  Ecchymosis.
General Indisposition.
Debility.
  Marasmus.
Cachexia Africana.
Diarrhœa, Dysentery.
Dislocation.
Fracture.
Disease of the Hip-joint.
Swelled Limbs.
  Anasarca.
Elephantiasis.
Varicose Veins.
Partial Atrophy.
Skin Diseases.
  Alopecy.
  Urticaria.
  Psoriasis.
  Impetigo.
Polypus of the Nose.
Prolapsus Ani.
Hæmorrhoids.
Jaundice.
Pneumatosis, Emphysema.
Hernia.
Hydrocele.
Scurvy.
Gonorrhœa.
Apoplexy.
Nephritis, Excretion of Calculi, Gravel, and Alteration of the Urine.
Excretion of Alvine Evacuations.
Ascites.
Opacity of the Cornea.
Cataract.
Varicocele.
Sarcocele.
Tetanus.
Hydrophobia.
Fasting.
Animals in the Stomach.
Animals in the Urine.
Vicarious discharge of Urine.
Suicide.
  Poisoning.
  Hanging.
  Drowning.
Death.

Having thus presented the bill of fare to the reader, we must refer him to the work itself for details. We can only afford space to refer very briefly to a few of the items.

*Pain* is a fruitful field for the cultivation of the malingerer. Here, however, a real sufferer, unless caution were observed, might appear inconsistent. The patient is not always able to specify the precise seat of the pain. Or its real seat may be mistaken by himself and the medical attendant. Thus, scirrhus of the stomach has passed for liver disease. Is there not always a sallowness of complexion in scirrhus of the stomach? The precise seat of pain in some cases of inflammation of the liver terminating in abscess, is sometimes not easily discovered. Watch the suspected simulator when he is asleep, (and of course completely off his guard,) advises our author. This indeed is a capital precaution in all instances of supposed simulation. There is much reason to apprehend that many sufferers from neuralgic pains have been very unjustly suspected of feigning, and been consequently put to serious inconvenience. A malingerer will be apt not only to shift his pains, but to exaggerate his acting. If written notes are regularly kept of a malingerer's pains and sensations, he will most likely, at some time or other, be found tripping. He who wishes to have accurate recollections of the physiognomy of pain should watch those who really suffer. Every emotion and expression of countenance or voice is wrung with difficulty from real suffering.

There is a fluency, a flexibility, and an impromptuness of groan, look, and spasm about the sham-Abraham, more easily recognised by him who has compared the true and the fictitious in the school of experience, than capable of being described in writing.

*Nyctalopia,* or night-blindness, is a disease of which but little is comparatively heard in Europe. In the East it is very common among all classes. Sepoys have often, to their own great inconvenience, to fall out of the ranks, and be led by a guide on account of it. Among them we have never met a simulated case.

*Amaurosis.*—"*There is in the eye and gait of the amaurotic patient an air of uncertainty; he has a staring unmeaning look.*" Impostors avoid things placed in their way. The effects of belladonna are now well understood by the vulgar for purposes of simulation.

*Myopia,* our author slyly observes, "is a state of vision which is very commonly assumed by society in general, when they wish to avoid the sight of an object that is disagreeable, or a friend that is objectionable."

*Deafness* originates a remark that is a pendant to this. "It is," we are told, "occasionally assumed in our courts of justice, and like Myopia, is a common resource of society to avoid hearing anything unpleasant." It may be added that it is sometimes resorted to for the purpose of hearing more than the speakers might bargain for if they did not believe the listener deaf. Cadwallader, Crabtree, and Sir Mungo Malagrowther has each a purpose in his deafness, representing a class thus delineated by the hand of a master, though differing in style. Those who pretend to *deaf-dumbness* have truly, according to our author, a very arduous task to play. The knaves may perhaps be literally caught napping, or, in other words, talking in their sleep. Women, Fodere says, have been the most successful simulators of deaf-dumbness. What heroism! What wonderful resolution to pass, even for a day, for deaf and *dumb!*

*Insensibility.*—The remarks under this head are a fine instance of the practical application of the principles of inductive reasoning. The following anecdote shews how the malice of a wretch may bring people into trouble.

"A servant, on receiving a trifling injury from her master, a clergyman, ran to the door, said she had been almost murdered, and to add strength to her assertion, pretended to fall into an epileptic fit. She was carried as one expiring to an hospital, and lay for ten or twelve days, without showing the least sign of sense or recollection. The clergyman and his wife were dragged to gaol—popular indignation ran high, and his property was destroyed; terror, and shame of such a public exposure, brought on an illness which placed his life in imminent danger, and greatly injured his fortune. Mr. Dean, on being called in to consultation, soon detected the imposture, and the woman almost immediately disappeared." 108.

A state almost amounting to syncope may come on, suddenly depriving a man of the power of speech and movement, but not of consciousness.

It may last only a minute or so, and yet overtake one during the performance of a responsible duty, as while a man stood sentry. There is reason to believe that people have been involuntary witnesses of crimes of the darkest kind, but have been deprived of the power of giving aid or alarm, by a catalepsy of terror. Somnolency at times is very overpowering, and sleep occasionally is so profound, that it is difficult to waken the person. We remember a medical man, who was so overcome with sleep after a hot night at Cawnpore in India, that an officer who ran to call him to give his assistance in a case of accident at a firing parade, did not succeed in rousing him. But for the friendly consideration of the officer, the Medico might have had to defend himself before a court-martial. It was a case of torpid exhaustion from heat. The party in general slept very lightly, and when on this occasion he awoke, was horrified at finding that all efforts to rouse him at a call of duty had been in vain! *Somnambulism* is not likely to be much simulated by soldiers or seamen. He who has once well observed the blank expression of the somnambulist's face, and the ghastly character of the open and half-open unspeculating eyes, and heard the hollow tones of his voice, with the blurred articulation (so to say) so different from his ordinary tones, and attends to the rhythm of respiration, is not likely to be imposed upon by a pretender.

*Vertigo and Cephalalgia* are frequently simulated. The most intolerable of all headachs would seem to be the cerebellar and basilar. A frequent symptom is a feeling as if the patient would fall backwards, but for an exertion against the tendency. In all severe headaches there is less or more drooping of the upper eyelid, and a peculiar heavy expression of the eye itself—there is also a frown or congestion of the brows. In bilious headach, a drawing down of the angles of the mouth may be observed; it is a movement of incipient nausea; there is also a redundancy of saliva.

*Hysteria.*—The appearance of symptoms simulating such an affection in a soldier would excite the utmost suspicion. "We might almost," our author thinks, "as readily expect to find him suffer the pains of labour or the effects of protracted suckling." We are not at issue with this as a general remark, but to deny that a man may be attacked with a train of symptoms either constituting a genuine hysteric paroxysm, or something very like it, would not be accurate. We use the term hysteric, of course, in a sense different from its etymology, for want of a more expressive one, just as we might speak of a masculine woman. In one of the instances of male hysteric affection the subject was a brother officer, a fine manly fellow to boot. He had been suffering previously from dyspnœa caused by contusion and concussion of the chest from a heavy fall. This was followed by convulsive movements and alternate fits of laughter, sobs, and tears, ending in apparent insensibility. Hartshorn was administered with beneficial effects. The person at the time was labouring under considerable mental depression; there was the sense of globus, and all idea of simulation was quite out of the question. As respects the fair sex, Dr. Conolly urges with great truth that unhappy temper and violent irritability of hysterical females, combined with their constitutional tendency to the hysteric paroxysm, is in some instances sufficient to bring on, almost at the will of the patient, attacks which occasion much concern to their relatives or friends; while Dr. Copland has no doubt of the fit being often repeated at pleasure, almost as readily as tears may be shed, by recalling or adverting to various feelings and emotions. There is no warning we would more emphatically impress upon young females than to resist with all their strength and resolution the first hysterical tendencies as they would the foul fiend. Young and foolish women are as anxious sometimes to commence their hysterics, as boys are to sport coat-tails or an imperial. The thing indicates promotion, and advancement in social importance. First comes a desire to interest the feelings of beholders especially of the other sex, and when anything crosses the young beauty's will and pleasure, we have a fit. The heroines of novels always have fainting or other fits, and why should not she? It is *so* interesting! It is aristocratic into the bargain. No doubt this sort of exhibition, when well conducted, *will* interest, and it is so delicious to excite interest! Men have sacrificed their lives to the passion of exciting interest. We need not rummage the pages of antiquity, for examples, like Peregrinus, or Empedocles. Modern Paris will furnish us with plenty of examples. Alas! the interest of hysteria rapidly wanes. That which was produced at will to serve some trivial purpose, becomes at length a confirmed habit of the constitution—that clings to the pure heroine as obstinately as the old man of the sea did round Sinbad's neck. If a woman who has literally worked her own nervous system into this wretched state of excitability could but have a correct notion of how great a bore (no other word will express it so well) she becomes to her own friends, to say nothing of society, and how her presence in the circle is dreaded as a sort of Banquo's ghost, that must produce " most admired disorder"—she would at least exert her will, and make an effort to get out of the meshes of the tyrant. We beg not to be misunderstood, for these remarks apply not to genuine and unavoidable disease, for which we must ever feel sincere sympathy, but to that which commences in wilfulness and affectation. Satisfied as we are that many cases might quietly evaporate without being obtruded on general notice, and that much of the liability to this spurious affection is fostered by injudicious sympathy, we would earnestly counsel a reserve in the expression of such. This may appear harsh, but we say it advisedly, and with a conviction of the real sufferings to which such exhibitions will eventually lead. We would fain exorcise the demon from the parlour, drawing-room, and boudoir. We record our convictions on this head with a deep sense of the high dignity of woman as man's helpmate, no less than of the misery she occasions when she swerves from the honest grace of her nature in striving to produce effect by means wholly unworthy of her, and to captivate us by storm, not by means of her native charms, but through the turbid media of wild emotion and spasm. Let not the first attacks of hysteria, then, in the young and strong be looked upon with mistaken tenderness: harsh as the advice may sound, let salutary austerity rather mark the demeanor of friends. Let the thing, if alluded to at all, be spoken of as what might have been prevented by the power of the will. This will set the patient upon her mettle to resist the next occasion as much as she can, and the habit of resistance may thus complete a cure. Speedy recovery will sometimes follow declarations made to surrounding friends in presence of the apparently unconscious patient. We remember a case where a poor fellow was almost driven distracted by the constantly recurring hysterics of his wife. Somehow a little matrimonial huff always preceded the exhibition. When called for the first time to her aid (for she had been long under the care of another practitioner) we could not help suspecting that at least three-fourths of the affection might be placed to the account of simulation. Having gone through the usual preliminaries, we expressed a conviction, that throwing a tub of cold water over the patient just as she lay, clothes and all, in bed, would immediately restore her to sensibility. A large tub was accordingly brought to the bed-side, and pailfuls of the cold element were poured in. This was sufficient; she opened her eyes and sat up quite restored! The bath was never required, though in her hearing we gave strict injunction to her husband, always to have recourse to it when she was so affected. Even where the affection may be genuine, but consciousness not wholly gone, some manœuvre of this kind may produce a good effect.

*Mania, &c.*—All between pages 121—176 is a very valuable contribution to medical literature. How vividly true these few descriptive words respecting the advent of insanity! "A change in the appearance of the eye precedes incoherence of language; there is a peculiar motion of these organs, *or protrusive and wandering motion peculiarly tiresome to behold.*" "The stools are *white*, small and hard;" this must have partly led the ancients to their conclusion that functional disease of the liver had much to do with insanity. During the war it seems that, amongst prisoners and those kept longer in the service than they desired, all the forms of disordered intellect were feigned. Most sincerely do we concur with Dr. Gavin in the opinion that, "a medical officer can never exercise too much caution in giving an opinion upon doubtful cases of mental disease, more especially where the opinion may involve a breach of discipline and consequent punishment." More frequently than may be generally supposed, simulation is suspected in mental disorder, and much injustice thus done to those who have a touching claim upon our commiseration. An officer, now no more, who served abroad, evinced symptoms of insanity. He was considered by some of his brother officers as shamming on purpose to get away from his regiment, and to be sent to England. The suspicion was cruel, as was proved by the event. On the voyage home he usually walked the quarter-deck, with a bugle horn suspended round his neck. One day when the sea was running high, they spoke a French ship which came close to them. This poor fellow, clad in a long great coat and military cap, walked out at the gangway into the sea, blowing his bugle horn. He was immediately carried a long way a-stern, and before aid could reach him he perished, blowing his bugle horn to the last, and buffeting the waves with his right hand! We would add to the caution another, in regard to hasty conclusions respecting intoxication. Persons staggering under nervous exhaustion, or excitement, have been accused of intemperance when there was no ground whatever for the aspersion.

No man should be pronounced a lunatic until after strict, careful, and, in some instances, repeated examinations by more than one. It may admit of doubt whether the examination is sufficiently formal at some of our colonies. In India we have heard that in more than one instance authority has decided the question of an official's insanity, mainly upon the opinion of a single medical officer. This is altogether wrong, and even suspicious, when the functionary may be in possession of an important and lucrative office, suspension from which might prove the ruin of himself and his family. It is no light thing to determine upon unsoundness of mind, and medical men may happen to be quite as ignorant of the philosophy of the subject as their neighbours. A mixed commission of medical and other officers ought always to be appointed for inquiry in alleged cases of insanity; and, as the affection may prove but of a temporary character, every facility should be secured for the patient being again and again brought before the committee. For a high authority to express an opinion upon such a case before the opinion of the committee was officially reported, we should hold not to be merely indiscreet but indecent. Simulators cannot feign the sleeplessness of insanity. We recollect the case of a young man in whom delirium tremens, brought on by drinking, terminated in insanity of rather a mild and jocular character. For six weeks previous to his death he never slept one wink. The most powerful narcotics in large doses failed to procure even an instant's repose. How terrific is this simple historic fact alluded to by our author—" Chiaggi saw a lunatic who had sat for twenty-five years on a stone floor, beating the ground with his chains, without leaning by day or night." There is nothing in the pages of the Inferno much more tremendous than this. Insensibility to pain and cold is one of the most striking phenomena of insanity. An insane man, for instance, will tear off his clothes when the weather is piercing cold. He will also, from the erect position drop suddenly on his bare knees upon the stone or brick floor of his cell, and repeat the feat several times, his knees coming with a force on the bare stones, that would smash a sane man's joints. The sense of smell is frequently depraved in the insane, and they are apt to fancy putrescent odours, and to attribute wild causes for their existence. Is it possible that this may be attributable to their perception of the maniacal odour in themselves? The hair and the skin have often a characteristic harshness and dryness to the feel. In simulators of sufficient cunning and resolution, feigned insanity is carried on when they are alone as well as when in the presence of others; as was shewn in the case of one of two brothers, who committed a high-way robbery near Edinburgh. One, on being brought up for trial, was found to be insane. The other was convicted and executed. The faculty of the modern Athens were divided in opinion respecting the survivor. The majority, if we recollect right, decided that he was insane. This excellent actor (for such he proved) being ordered to be discharged and sent to his friends, left the Tolbooth (it then existed) the day after the receipt of the order as sane as any man in Edinburgh. According to Ray, when the disease comes on suddenly, abruptly, and violently, and there be the least ground for suspicion, it may be safely concluded that the case is one of simulation. Genuine insanity, however, may come on in this sudden manner. A most estimable member of a family of rank, without the slightest premonitory sign, walked into the drawing-room, and struck his mother a violent blow on the face. He never recovered. The remarks of Pinel and others have shewn that a sanguinary instinct may be accidentally developed in the most virtuous man, and may carry him often irresistibly, without any reasonable motives, to the most terrible excesses. Other instincts may be similarly developed. It has been stated of the notorious duellist, "fighting Fitz-gerald," that, previous to a wound in the head, he was a mild and amiable man. Be that as it may, injuries of the head produce curious modifications of character, just as concussions of the body from gunpowder have been observed in some instances to have brought on obesity and a general stoutness. We were acquainted with two officers who attributed their extraordinary size after the event to the circumstance of being blown up, the one in Madras Roads, and the other in storming a fort. One person who received a severe concussion of the brain by a fall from his horse, became soon after capricious and cold to old friends, with whom he had been formerly most cordial. Another gentleman, who, up to his 40th year, had been noted for steadiness, respectability, and sobriety in his profession (the law), was severely wounded and concussed by a fall from his horse—and became an incurable drunkard. The recollection of such things ought to make us charitable towards our neighbours' failings. We know not what may be at the root of them! "Dr. Genget believes that madmen have died upon the scaffold." If Macnaughten was mad when he shot Mr. Drummond, what are we to think of Bellingham's execution? The criminal, we are told, has always a motive. What was the motive that caused Nicholson to murder Mr. and Mrs. Brown? Women have been hanged for child-murder who ought to have been sent to an asylum. Even some of the inferior animals have cerebral excitement or delirium after parturition, which leads them to destroy their young.

The pen of genius has given us a fine illustration of moral insanity in the character of De Montfort.* The actions of some of the Roman Emperors were those of madmen—Caligula, Heliogabalus, and Commodus, would, in our day, instead of being slaughtered, be consigned to the care of a keeper—but in that far olden time no one dared "bell the cat," and within the recollection of some among ourselves no more humane plan of restraining that madman the Emperor Paul suggested itself than the coarse one of a halter. Despots, therefore, have cause to dread insanity more than common men; for as old Caustic says, respecting his nephew Tangent, in the Comedy, "Give him plenty of straw—plenty of straw"—but with a poor Autocrat of all the Russias, when he becomes queer the rule is "give him plenty of *sash*." Of so much importance is the comprehensive subject of madness, in all its phases and varieties, that an academic chair ought to exist in every medical school, for teaching it as a distinct branch of science.

Of *Nostalgia*, we have seen several real but no feigned cases. Almost all who fell under our observation, were highlanders, and some of the Indian hill-tribes. In one case, the patient was a Frenchman from Brittany, who had not the means of returning to his native land. It terminated in insanity of a mild character, closed by death. Those affected with Nostalgia are reserved and wrapt up in melancholy contemplation. They do not willingly commence about the object of their yearning.

---

* Joanna Baillie's Plays of the Passions.

*Epilepsy.*—In detecting its simulation, this remark of an author should be borne in mind—" the false shame of true epileptics, and the want of shame of feigned ones, has been taken notice of by several writers." Suggesting symptoms is an excellent trap in this and other simulations. We cannot approve of the employment of " seven or eight drops of croton oil," for the detection of imposture, as used by a Mr. R. With many it might lead to much more serious results than running to the water-closet. In all doubtful cases, the practitioner should take the side of mercy. The following remarks on Hemiplegia are of practical value.

" In suspicious cases of hemiplegia, our inquiries must embrace the origin of the attack, its nature and course. Whether arising 1, from apoplexy; 2, or likely to precede it; 3, whether characterised by previous symptoms, such as pain in the head, disorder of the intellectual powers, spasmodic twitchings; 4, whether gradually supervening in persons in advanced life; 5, whether preceded by a train of anomalous and perplexing symptoms, having a relation to chorea, or fits of an epileptic character; or, 6, whether succeeding at some period after the receipt of an injury.

" In the attack itself, if with the loss of voluntary power over the upper and lower extremity, we do not recognise paralysis of the side of the face, a drawing of the mouth to the sound side, more or less upwards; a curve of the tongue when protruded, the convexity being towards the affected side; an increased dilatation of the nostril of the sound side, which is not equalled by that of the paralysed, when a long inspiration is made; the peculiar pointing of the foot when it falls, as it were, by its own gravity; adduction of the affected arm, and slight flexion of the fore-arm, wrist, and fingers: we have every reason to believe the case pretended.

" In pretended hemiplegia, asserted to be the result of an injury to the head, the simulator is not likely to be aware that the paralysis should occur on the side opposite to that injured." 215.

For the benefit of our less experienced readers, some instances of detection may be mentioned.

" Malingerers pretending to have lost the use of their limbs, have been detected by putting them, without their knowledge, under the influence of opium, and tickling them when in profound sleep; or by binding the sound arm to the side, and irritating the nostrils during the night with a feather.

" They have betrayed themselves by using the limb on their first awakening, before they recollected themselves.

" A case is related where a man, pretending to have lost the use of the extensor muscles of the right hand, was detected by his gradually raising his arm as far as the extensor muscles could carry it, on the near and nearer approach of a red-hot poker.

" On an alarm of fire being given, an individual, who for two years had pretended paralysis of the lower extremities, and endured every thing that medical skill and suspicion could suggest, saved not only himself, but his trunk and clothes.

" One man was detected by rubbing his feet with cowhage (*Dolichos pruriens*). He walked and groaned all night, and next day reported himself fit for duty.

" Dr. Davis at Chatham, knocked gently at the dusk of the evening, on the window of one who could not move, and had lain in bed for a month. On calling him gently by name, he was at the window in an instant." 220.

In Smollett's Humphrey Clinker, Micklewhimmen is an exact prototype of the malingerer roused by the alarm of fire.

*Rheumatism.*—The Chronic, especially, is a favourite speculation of the malingerer. Our author's remark about "a look of delicacy," is a very important one, and we recollect not an instance of the genuine disease where this "look of delicacy," as it has been happily called, was absent.

*Enemesis.*—In endeavouring to detect its simulation, the course adopted by even so high an authority as Foderé, (page 235, )strikes us as objectionable. The application of moxa to the scrotum (page 236,) we hold to be an outrage.

*Lameness.*—Under this head occurs a very instructive case.

"A street porter, after a fall, began to complain of pain stretching along the whole outside of the thigh. The pain was much aggravated by motion, so that he could not walk across the yard without a crutch. The most attentive examination, scrupulously and laboriously made, could discover nothing deviating from the ordinary structure and appearance; nor was there any general affection of the system. The patient was the object of suspicion. It was a severe Winter —employment for porters was said to be scarce—the lodging and food of the infirmary were comfortable—and the alimony of a benefit society was accumulating in his favour. He readily submitted to the most violent counter-irritants, but without acknowledging any relief. The only remedy which relieved the pain being Perkins's metallic tractors (then in vogue) increased the suspicions previously entertained. He was dismissed from the hospital, with *simulation* affixed to his name on the records, and he was struck off from the rolls of the friendly society. Two weeks after his dismissal, he died of apoplexy. The thigh was inspected. The cartilage covering the head of the femur was partially destroyed, and purulent matter, to the amount of two ounces, was found in the cavity of the joint. This case occurred in the Infirmary of Edinburgh, about thirty years ago, and was under the care of Dr. Duncan, assisted by Dr. Bateman." 240.

The following extracts shew the ingenuity of malingerers.

"Fever, or rather febrile symptoms, may be induced by the use of various stimulants—as wine, brandy, cantharides, &c. Tobacco, whether taken internally or introduced into the anus, quickens the pulse, and produces an appearance of general indisposition. Hutchinson has found, by this drug, (in a simulated case,) the pulse small and rapid, accompanied by considerable emesis. I have been informed by Dr. Thompson, R.N., that he has seen this drug frequently made use of to simulate a paroxysm of fever.

"Foderé states that he has observed a feverish state of the system induced by violent exercise, which the authors of the *Cyclop. of Pract. Med.* have also seen used for the purpose of carrying on this fraud. A paroxysm of fever is said to be excited by the introduction of a clove of garlic into the rectum; and Zacchias says that the seed of henbane, when drank, excites fever, 'but it also excites the mind, and renders men frenzied.' Acrid kinds of food, and drugged spirits of wine, also produce this effect.

"The tongue, in order to imitate the appearance it presents in fever, has been covered with chalk, pipe-clay, tobacco, brick-dust, soap, flour, whiting from the walls, &c. The apparent bilious tinge of a coated tongue may be caused by chewing a little gingerbread.—It is always easy to detect this circumstance, by causing the patient to wash his mouth well with tepid water. The urine is rendered of a pale colour by dilution with water, or, of a highly ammoniacal odour, by long retention." 253.

Such a statement as the following, ought to put all medical officers on their guard.

"There are several instances on record, where a board of medical officers has recommended recruits to be discharged from the army, on account of alleged great deformity; who were, in fact, remarkably well-made men, and were afterwards re-approved." 267.

In our author's chapter on Hæmoptysis, occurs a most instructive case.

"A soldier, pretending illness, asserted that his complaint arose from blows received from the serjeant-major at drill, to whom he bore an ill-will; the fauces were slightly reddened; in a few days the throat became more inflamed, and he was utterly incapable of swallowing anything but liquids. This was followed by ptyalism; he soon began to spit blood of a slight scarlet colour, but without cough, this increased in quantity daily. In a short time he was observed to be constantly spitting or hawking up blood, and became very white and emaciated, till, in a day or two, sudden hæmorrhage carried him off. On opening the body, Mr. Guthrie found an instrument lying across the commencement of the œsophagus, composed of two half phial corks, fastened together by a strong thread, having previously each had three pins thrust through them, so that the heads of the pins were applied to each other back to back, the points sticking out beyond the cork, forming a sort of chevaux-de-frise; this, it is presumed, he covered with fat, and attempted to swallow, but, the point of a pin catching, the efforts to swallow turned the machine across. In this situation, the points of the pins were close to the carotid arteries, and having, by degrees, given rise to ulceration of the œsophagus, they wounded them on both sides; every elongation or pulsation of the arteries having brought them against the point of one or more of the pins, the marks of which were observable in several small holes of different sizes on the sides of the vessel; these holes gradually increased by ulceration till they gave rise to the fatal hæmorrhage. The arteries and pins are in the possession of Dr. Hooper.

"I have been thus particular in the symptoms and history of this case, as it shows the difficulty of diagnosticating the cause of some factitious diseases, and consequently the difficulty of treatment; the man denied imposition to the last. Sir George Ballingall, in his Lectures, (1836-7,) mentioned that a pin accidentally swallowed produced a similar result to that of the foregoing case. Sometimes impostors put into the mouth pastilles coloured with carmine, and prepared with acid substances, which excite the salivary secretion. Some pretend this disease by the aid of a small piece of Armenian bole placed beneath the tongue. Brickdust, and vermillion paint have also been employed for the same purpose. Beck quotes a curious case under the head of *cachexia,* which properly belongs to hæmoptysis. One Henry Moor Smith, a most accomplished villain, while in the prison at Kingston, began to split blood, had a violent cough and fever, and gradually wasted away, so that those who visited him supposed that his death was rapidly approaching. This continued for a fortnight, and his weakness was so great, that he had to be lifted up in order to take medicine or nutriment. A turnkey unfortunately, however, left the door of the prison open for a few moments, in order to warm a brick for his cold extremities; on his return, *Smith had disappeared.* On again being put into prison, he feigned cachexia, hæmoptysis and epilepsy, but with no success. He confessed that he pretended to raise blood by pounding a brick into powder, putting it in a small rag, and chewing it in his mouth. He contrived to vary his pulse by striking his elbows: and said he had *taken the flesh off his body in ten days by sucking a copper cent in his mouth all night and swallowing the saliva.*" 288.

Here, however, we must bring our remarks and extracts to a close. The work may be considered a standard one. We recommend it to such of our readers as desire to become acquainted with the impostures of malingerers.

### On Healthy and Diseased Bone.

