# Errata

**Published:** 1844-04-01

**Authors:** 


*Errata in the Article " Dr. Gavin on Feigned Dieases," in No.* 79, *Jan.* 1844, *Medico-Chirurgical Review.*

Page 72—3rd line from top, for "*there* were six lives lost," read "here were six lives, &c."—Page 75—10th line from top, for " evening rounds of his *side,*" read "evening rounds of his sick."—Page 79—17th line from top, for "the open *and* half open eyes," read " open or half open."—Page 79—26th line from top, for "frown or *congestion* of the brows," read "frown or corrugation."—Page 83—24th line from top, for "what was the motive that caused Nicholson to murder Mr. and Mrs. *Brown,*" read "to murder Mr. and Mrs. Bonar."

